# Small World derived index to distinguish Alzheimer’s type dementia and healthy subjects

**DOI:** 10.1093/ageing/afae121

**Published:** 2024-06-27

**Authors:** Fabrizio Vecchio, Francesca Miraglia, Chiara Pappalettera, Lorenzo Nucci, Alessia Cacciotti, Paolo Maria Rossini

**Affiliations:** Brain Connectivity Laboratory, Department of Neuroscience and Neurorehabilitation, IRCCS San Raffaele Roma, 00166 Rome, Italy; Department of Theoretical and Applied Sciences, eCampus University, Novedrate, Como, Italy; Brain Connectivity Laboratory, Department of Neuroscience and Neurorehabilitation, IRCCS San Raffaele Roma, 00166 Rome, Italy; Department of Theoretical and Applied Sciences, eCampus University, Novedrate, Como, Italy; Brain Connectivity Laboratory, Department of Neuroscience and Neurorehabilitation, IRCCS San Raffaele Roma, 00166 Rome, Italy; Department of Theoretical and Applied Sciences, eCampus University, Novedrate, Como, Italy; Brain Connectivity Laboratory, Department of Neuroscience and Neurorehabilitation, IRCCS San Raffaele Roma, 00166 Rome, Italy; Brain Connectivity Laboratory, Department of Neuroscience and Neurorehabilitation, IRCCS San Raffaele Roma, 00166 Rome, Italy; Department of Theoretical and Applied Sciences, eCampus University, Novedrate, Como, Italy; Brain Connectivity Laboratory, Department of Neuroscience and Neurorehabilitation, IRCCS San Raffaele Roma, 00166 Rome, Italy

**Keywords:** Alzheimer’s, graph theory, electroencephalographic (EEG), neurorehabilitation, Small World, older people

## Abstract

**Background:**

This article introduces a novel index aimed at uncovering specific brain connectivity patterns associated with Alzheimer's disease (AD), defined according to neuropsychological patterns.

**Methods:**

Electroencephalographic (EEG) recordings of 370 people, including 170 healthy subjects and 200 mild-AD patients, were acquired in different clinical centres using different acquisition equipment by harmonising acquisition settings. The study employed a new derived Small World (SW) index, SWcomb, that serves as a comprehensive metric designed to integrate the seven SW parameters, computed across the typical EEG frequency bands. The objective is to create a unified index that effectively distinguishes individuals with a neuropsychological pattern compatible with AD from healthy ones.

**Results:**

Results showed that the healthy group exhibited the lowest SWcomb values, while the AD group displayed the highest SWcomb ones.

**Conclusions:**

These findings suggest that SWcomb index represents an easy-to-perform, low-cost, widely available and non-invasive biomarker for distinguishing between healthy individuals and AD patients.

## Key Points

- Electroencephalographic (EEG) Small World (SW) organisation in brain networks is examined for potential insights into neurodegeneration in conditions like Alzheimer's disease (AD).
- A derived SW index as a reliable biomarker for early diagnosis and risk stratification.
- AD affects brain's network architecture, emphasising the importance of understanding the complex brain organisation in ageing.

## Introduction

Alzheimer’s disease (AD), the predominant type of dementia, affects various cognitive domains [1] and imposes an enormous burden on patients and healthcare systems [2].

Neurodegeneration disrupts the brain's network architecture leading to cognitive decline and brain damage [3]. Understanding the organisation of the human brain as a complex network has emerged as a powerful framework to study ageing and neurodegeneration processes.

Over the past two decades, the application of innovative mathematical approaches such as graphs theory on electroencephalographic (EEG) data has experienced a significant increase [4, 5]. The graph theory allows describing the brain through a mathematic model, the graph, which provides a simple representation of a complex system. The graph is a network model consisting of nodes, representing sets of neurons, and edges, which are the connections existing between the nodes. The characteristics of the graph are measurable through several parameters, such as the Clustering Coefficient, which represents the number of connections that exist between the nearest neighbours of a node as a proportion of maximum number of possible connections and reflects the tendency of a network to form topologically organised circuits, or the Path Length, the minimum number of edges that must be traversed to go from one node to another.

In particular, a key discovery highlights the Small World (SW) organisation existence in human brain networks. The SW index, assessed on brain networks, represents the organisation of the brain network as it estimates the balance between *segregation*, local connectedness, and *integration*, global connectedness, of a network supporting efficient information transfer as well as some local specialisation [6]. The small-worldness measure of the network was determined by the ratio of the normalised clustering coefficient, representing the network's ability to create separate clusters, to the normalised path length, the network's ability to become interconnected and exchange information.

This dynamic structure exhibits significant alterations in neurological disorders [7] suggesting that SW might unravel the neural dynamics of neurodegeneration and possible changes in connectivity in conditions like AD. Therefore, over the years, as AD has been increasingly considered as a synaptic disconnection syndrome, its complex brain dynamics have been studied with the network approach.

Previous research explored network differences to aid the early diagnosis of dementia and distinguish AD patients from healthy individuals [8]. In particular, SW index values seem to increase in the alpha band [9] and to decrease in delta, theta and beta bands [10–12] in AD patients compared to healthy subjects. Furthermore, studies reported a significant reduction in the SW of mild AD patients in all the EEG frequency bands compared to healthy controls [13, 14]. Regarding the conversion to AD, Miraglia *et al.* [15], studying SW in the Default Mode Network, discovered that SW index in the gamma band decreased in converted MCI subjects compared to stable MCI subjects. Moreover, in converted MCI subjects with impairment in linguistic domain, the SW index significantly decreased in the delta band, while in those converted MCI subjects with impairment in the executive domain, the SW index decreased in the delta and gamma bands and increased in the alpha 1 band. Furthermore, several studies have highlighted correlations between SW index with neuropsychological tests and other AD biomarkers. In particular, a correlation between SW in beta and gamma bands and the deposition of the protein tau has been reported [16], meaning that the higher tau burden in early AD’s disease was associated with a shift away from the optimal SW organisation and a more fragmented network, especially in the beta and gamma bands. Tait and collaborators [17] found a positive correlation between SW calculated in the temporal lobe and the language sub-score of mini-mental state examination (MMSE), indicating that disruption in temporal lobe connectivity plays an important role in the language impairments of AD subjects.

Based on these results, it is evident how the SW index succeeds in representing networks dysfunctions induced by AD and in distinguishing the organisation of pathological networks from healthy ones. Moreover, it is evident that these changes occur differently in the distinct frequency bands of the EEG (delta, theta, alpha 1, alpha 2, beta 1, beta 2 and gamma). Following these lines, we believe that considering only one SW based index, incorporating all the EEG frequency bands, rather than considering frequency bands separately represents most effectively all the changes brought by the pathology.

Therefore, this study presents an innovative approach that proposes a new derived SW EEG index, incorporating information of the seven EEG rhythms, aimed to identify specific patterns that differentiate healthy ageing from AD, which could potentially serve as a reliable biomarker for early diagnosis and risk stratification.

## Subjects and methods

### Subjects

A total of 170 healthy subjects (55.3% females; mean age = 71.07 years, Standard Error (SE) = 0.45y) and 200 patients with mild-AD (65% females; mean age = 72.32 years, SE = 0.49y) were included. Participants were recruited from a wide range of Italian facilities including hospitals, Institutes of Scientific Research and Healthcare (IRCCSs—Instituti di Ricovero e Cura a Carattere Scientifico) and research centres. Demographic and clinical information are provided in Table 1. No significant correlations between demographic data and the derived SW index were found. This analysis strengthens the robustness of our findings and underscores the reliability of the SW_comb_ index as a measure unaffected by demographic factors within the range considered.

**Table 1:** Demographic and clinical characteristics of the three groups enrolled in this study, healthy and AD patients. The table includes information on years (y) of age and education, and MMSE scores for each group in terms of mean values and SE

	***Age***	***Education***	***MMSE***
*Healthy*	*71.07y ± 0.45y*	*10.78y ± 0.33y*	*28.37 ± 0.135*
*AD*	*72.32y ± 0.49y*	*8.36y ± 0.34y*	*20.54 ± 0.276*

Recruitment criteria for healthy subjects included no history of neurological or psychiatric disorders, or current vasoactive or psychotropic medication treatment.

The mild-AD diagnosis was established according to the National Institute on Aging-Alzheimer’s Association workgroups [18] and DSM IV TR criteria [19] and based on neuropsychological testing.

In this article, we have exclusively focused on mild AD patients, primarily due to their representation of the initial phases of the disease. During these stages, patients typically exhibit higher levels of autonomy and cooperation, making them suitable candidates for EEG recordings conducted for research purposes. Additionally, our research interest lies in delving into the distinct characteristics of brain networks during the early phases of AD. We aim to potentially expand this analysis to include the prodromal stage (MCI) in the near future. Informed consent was obtained from each participant, adhering to the Code of Ethics of the World Medical Association (1997) and following the principles outlined in the Declaration of Helsinki.

### Data recordings and preprocessing

Brain activity was recorded from each subject through EEG recording for at least 5 min in eyes-closed rest condition. The EEG recordings were obtained from different clinical centres (hospitals, IRCCSs and research centres) using different equipment, with at least 19 wet electrodes placed according to the International 10–20 system, after an acquisition procedures harmonisation. Vertical and horizontal electrooculographies (EOGs) were used for eye movements and blinking. Impedance was kept below 10 KΩ and the sampling rate was at least 256 Hz. The data were processed in Matlab R2022b using scripts based on EEGLAB.

All recordings were down-sampled to 256 Hz and were band-pass filtered from 0.2 to 47 Hz using a finite impulse response filter. The imported data were divided into 2 s epochs, and records were cleaned. Regarding the artefact cleaning procedure, our pipeline involves two main steps. Initially, we manually inspect epochs to identify and remove those containing isolated artefacts within the epoch itself. Following this inspection, we apply the Infomax Independent Component Analysis (ICA) algorithm. The ICA algorithm is a powerful technique used to separate mixed signals into their constituent sources. In our study, it helps us effectively remove various types of artefacts that are distributed throughout the EEG recording. For instance, in conditions where participants have their eyes open, as in the present experiment, we can efficiently remove blink artefacts using ICA. Similarly, if a channel exhibits high-frequency artefacts consistently throughout the experiment, ICA enables us to clean that channel by removing the artefact and reconstructing the underlying brain activity. Additionally, ICA is instrumental in addressing artefacts such as cardiac interference, which can occur if a channel is positioned near a blood vessel. This type of artefact typically affects the entire EEG recording, but with ICA, we can successfully mitigate its effects. Each type of artefact exhibits distinct power spectra and characteristic topographies, facilitating their identification and removal using ICA. Overall, the combination of manual epoch inspection and ICA-based artefact removal ensures robust data quality by effectively eliminating various types of artefacts while preserving the integrity of the EEG signals.

### Functional connectivity analysis

Connectivity between 84 regions of interest (ROIs) based on the 42 Brodmann areas available for both hemispheres [20, 21] was assessed using the eLORETA software.

The intracortical Lagged linear connectivity was calculated for all possible pairs of the 84 ROIs within each of the seven EEG frequency bands, namely delta (2–4 Hz), theta (4–8 Hz), alpha 1 (8–10.5 Hz), alpha 2 (10.5–13 Hz), beta 1 (13–20 Hz), beta 2 (20–30 Hz) and gamma (30–45 Hz), for each subject.

### Graph analysis

The graph analysis represents the network as a complex system consisting of nodes (vertices) and links (edges) connecting nodes.

Weighted and undirected brain networks were constructed using the Brain Connectivity Toolbox, adapted into Matlab scripts. The cortical sources estimated in the Brodmann areas served as vertices, and the edges were weighted using the Lagged linear connectivity values between each pair of vertices.

The SW index was determined by calculating the ratio of the normalised clustering coefficient to the normalised characteristic path length for all EEG frequency bands [20].

### Small world derived index (SW_comb_)

AD altered all the EEG frequency bands. A suitable approach appears to be the combination of the seven parameters into a single index instead of selecting a single band or a subset. COBYLA (Combined by Linear Approximations), used to find the optimal combination of parameters, was applied to combine the seven EEG parameters into a single index capable to maximise the discriminative power between AD and healthy subjects.

COBYLA iteratively explores different combinations of feature weights while adhering to predefined constraints, such as the need to preserve the EEG original information. This allows the creation of a composite index that enhances the accuracy, sensibility, specificity and effectiveness of AD diagnosis.


\begin{align*} {SW}_{comb}=&\ {w}_{\delta}\ast{SW}_{\delta }+{w}_{\theta}\ast{SW}_{\theta }+{w}_{\alpha 1}\ast{SW}_{\alpha 1}+{w}_{\alpha 2}\\&\ast{SW}_{\alpha 2}+{w}_{\beta 1}\ast{SW}_{\beta 1}+{w}_{\beta 2}\ast{SW}_{\beta 2}\\&+{w}_{\gamma}\ast{SW}_{\gamma } \end{align*}

### Statistical analysis

The aim of this study is to investigate whether the SW_comb_ index differs in healthy subjects and AD patients. Subsequently, a one-way analysis of variance (ANOVA) with the factors Group *(*healthy, AD*)* was conducted with a significance level of 0.05 using Statistica v.8 (StatSoft Inc).

A machine learning approach was used to quantify the SW_comb_ parameter ability in distinguish healthy and AD groups. A fine Gaussian Support Vector Machine (SVM) was employed using the SW_comb_ as a feature. A 5-fold cross-validation approach was used to optimise the SVM parameters. Moreover, in order to have an exhaustive evaluation of the classifier's performance, the 5-fold cross-validation was iterated 100 times. The accuracy is reported as the mean of accuracy for all the iteration and to assess the variance between each fold the standard deviation was also computed. The same approach was used to evaluate the specificity, sensitivity, while Area Under the Curve (AUC) and the Receiver Operating Characteristic (ROC) curve of the best classifier were represented in Figure 2.

**Figure 1:**
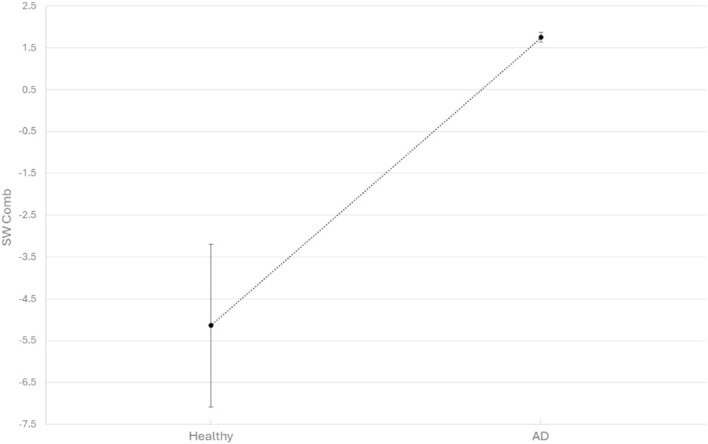
SW_comb_ indexes (mean values and SE) in healthy and AD groups are reported.

**Figure 2:**
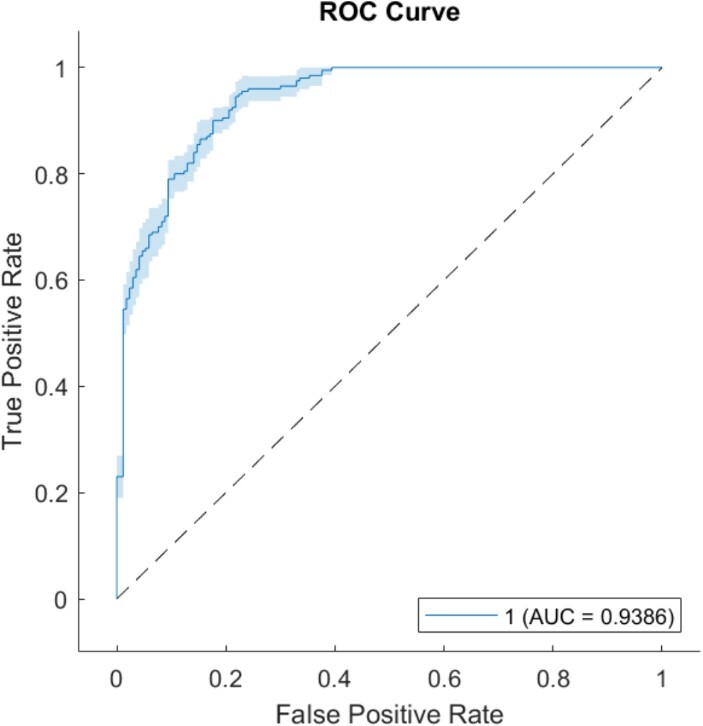
The ROC curve and AUC for Alzheimer's vs. healthy individuals best classification are represented.

## Results

The weights obtained using COBYLA are as follows:


\begin{align*} {w}_{\delta }=&-1.74840277;{w}_{\theta }=-16.95099133;{w}_{\alpha 1}\\=&\,0.22804703;{w}_{\alpha 2}=0.31640351;{w}_{\beta 1}\\=&\,4.88293561;{w}_{\beta 2}=0.87371046;{w}_{\gamma }=4.82353699. \end{align*}

The mean and SE of SW_comb_ index, reported in Figure 1, are −5.13945 ± 1.942736 for healthy and 1.753125 ± 0.122933 for AD group. The ANOVA revealed a statistically significant interaction (F(1, 370) = 14.734066, *P* = 0.000145683), indicating that the two groups exhibited distinct SW_comb_ values, demonstrating that the healthy group displayed lower SW_comb_ values compared to the AD ones (*P* = 0.000145683).

Furthermore, the partial eta squared of the ANOVA analysis was computed, resulting in a value of 0.038. This value represents a medium-average effect size based on the values defined by Cohen.

Regarding the classification between AD and healthy groups using the SW_comb_ parameter, the SVM reached an accuracy of 87.027% ± 1.99, a sensibility of 95.5% ± 1.57, a specificity of 77.1588% ± 2.86 and an AUC of 0.9386. The classification ROC curve and the AUC of the best classifier are shown in Figure 2.

## Discussion

The present study aimed to investigate the potential of a novel index applied to EEG distinguishing healthy ageing from AD.

The SW_comb_ serves as a comprehensive metric designed to integrate essential information from all seven SW EEG parameters, computed across the typical EEG frequency bands, creating a unified index that effectively distinguishes individuals with AD from healthy ones.

Results revealed a statistically significant interaction between the two groups that exhibited distinct SW_comb_ values. Specifically, the healthy group displayed the lowest SW_comb_ values, while the AD group had the highest.

The discriminative capability of this index in distinguishing AD from healthy individuals was validated through the SVM achieving an accuracy of 87.027% ± 1.99, a sensitivity of 95.5% ± 1.57, a specificity of 77.1588% ± 2.86 and an AUC of 0.9386.

This index is born from the assumption that the AD is characterised by alterations across various frequency bands. Delta and theta rhythms reflect interruption of connectivity and ‘isolation’ of local neuronal assemblies [22] in healthy subjects, and AD present an increased prevalence and amplitude of these rhythms, even during wakefulness [4]. Conversely, the alpha rhythms experience a power reduction in AD [4]. Additionally, beta rhythms, associated with active cognitive processes and attention, present a power decrease in AD [23]. Gamma rhythms, associated with complex cognitive functions, manifest changes in amplitude and coherence in AD [24].

Studying these alterations from the perspective of network analysis and graph theory provides valuable insights into AD brain connectivity.

Vecchio and colleagues [21, 25] have already demonstrated a SW pattern, assessed in healthy elderlies and AD patients, presenting a healthy>AD trend in the low frequencies and the opposite trend in the alpha band. The reduction of SW index within the low-frequency bands in AD patients was explained as a gradual decline in the communication/information flow among brain regions, indicating a shift towards a more ordered brain structure. In dementia, this decline in slow activity can be primarily attributed to the structural and functional deterioration of long corticocortical tracts [25].

In contrast, the alpha rhythms displayed a more random organisation (higher values of SW) in AD compared to healthy subjects. This increased randomness in the patients' network suggests a diminished cognitive efficiency, resulting in a reduction of cortical network performance [12]. Altogether, prior studies speculated that the lower SW values in low-frequency bands and higher SW values in alpha may indicate a functional disconnection [26].

Concerning the beta band, AD was distinguished by an extended characteristic path length, accompanied by a relative preservation of local clustering [27].

We proposed the SW_comb_ as a comprehensive index incorporating information of the seven EEG rhythms, with the aim of outlining the distinctive EEG characteristics associated with AD.

In conclusion, this study represents an important step towards identifying reliable biomarkers for AD early diagnosis, allowing timely and personalised interventions, and distinguishing AD from other forms of dementia and healthy ageing.

## Data Availability

The data that support the findings of this study are available on request from the corresponding author.
